# Development of narratives in Tamil-speaking preschool children: A task comparison study

**DOI:** 10.1016/j.heliyon.2021.e07641

**Published:** 2021-07-21

**Authors:** Krupa Venkatraman, V. Thiruvalluvan

**Affiliations:** Annamalai University, Annamalai Nagar, Chidambaram, Tamil Nadu, India

**Keywords:** Narratives, Utterances, Microstructure, Cognition, Story retell and Generation

## Abstract

‘Narrative’ can be simply defined as a spoken or written account of connected events or experiences. The present study records the development of microstructure elements of narratives in 200 typically developing Tamil-speaking children aged between three years and six years and eleven months. It then compares their narrative productivity across two elicitation contexts: story retelling (SR) and story generation (SG). The samples thus obtained are analyzed for three narrative microstructure parameters, namely total number of words (TNW) in the narrative, mean length of utterances (MLU) and the number of utterances. The results reveal an increasing trend in all three microstructure parameters across both contexts. All three parameters are found to be quantitatively high in SR than in SG. Variation in the performance in these narrative tasks has been explained with behavioural observations from literature, cognitive architecture and a working memory model. It was found that gender differences do not follow a uniform pattern across age groups and elicitation contexts. Since the study has generated normative data for microstructure parameters of narratives, the observations can be used to analyze language deviance and help plan the narrative intervention protocol for language therapy.

## 1 Introduction

‘Narrative’ is the earliest form of a monologic discourse used as a means to report, analyze and regulate daily activities (Ukrainetz, 2006). As a form of storytelling, narratives is an integral part of human tradition and culture, passed down through generations since historic times.

Feagans and Short (1984) argue that narrative is one of the most critical dialect capacities for school success. Narratives are useful in understanding the development of oral language and conceptual development and have a predictive function in assessing literacy and academic success in children (Stadler and Ward, 2006).

Narrative skills are universal to all languages and cultural groups. Language-based anthropological studies, examining the role of narratives in a socio-cultural context, suggest the need to evaluate narratives in every language across all genres (Heath, 1986). There are often cultural differences in storytelling across languages groups. Certain language groups exhibit a monologic style, while others incorporate conversational ways. Sethuraman and Smith (2010) conducted a cross-linguistic study analyzing the verb argument in scene description task using pictures and movies that depicted Tamil- and English- cultures in Tamil- and English-speaking adults and children. They report that children speaking Tamil often leave out certain objects and verb arguments while describing scenes or pictures depicting the English culture.

The genres and style of storytelling across language groups vary. Thus, it is crucial to analyze and record the norms for every language to account for this variability (Johnson, 1995). Although extensive research on multiple parameters of narrative microstructures has been conducted for languages such as Finnish, Swedish, and English, the generalizability of these studies is questionable in clinical assessments (Pankratz et al., 2007). Variations are reported in the presentation of narrative microstructures across different languages and it is crucial to develop normative data in a language for the purpose of assessment and intervention planning (Justice et al., 2006; Rollins et al., 2000).

Narratives are culturally and linguistically sensitive in that the pattern of a narrative organization could vary between languages. Narrative discourses occur in all societies and reflect the teller's culture; it also has certain universal characteristics (Ukrainetz, 2006). Researchers suggest that the narratives of children vary based on their culture and language (McCabe, 1997). These variations mark the need to establish language-specific norms for narrative measures of neurotypical children.

### 1.1 Narrative elicitation procedures (NEP): story generation versus story retelling

‘Narratives’ are complex organized verbal recounts that start from a highly contextualized environment and reach decontextualized generated events. The literature review below explains the narratives types, narrative elicitation procedures and the microstructure measure of narratives.

The narrative skills of children originate from their recounting of daily life events. The literature proposes three major ways of eliciting narratives, namely personal narratives, story retelling (SR), and story generation (SG). Despite the variety of narrative forms, the way story is elicited has a significant impact on its structure, productivity, and complexity (Duinmeijer et al., 2012). Two major narrative elicitation procedures used in the literature are SR and SG (O'Neill et al., 2004; Westerveld and Gillon, 2010b). Both the procedures use fictionalized narratives as stimuli. Fictionalized narratives have been of interest in elicitation as it potentially reveals formal narrative performance as compared to informal conversational narratives (Hughes, 2001; Ukrainetz, 2006). Using structured fictionalized narratives in evaluation shows variability in performance across age groups (Ukrainetz, 2006).

Allen et al. (1994) noted that children expressed a lot of content and action sequences in fictional stories. Terry et al. (2013) additionally found statistically important variations in personal and fictional narratives' microstructure measures. Thus, children's storytelling skills dissent across narrative genres. Shiro (2003) argues that different genres of narratives develop at different paces. In personal narratives, the context would be restricted to the observer and tend to make the scoring and analysis tough. Younger age groups might make personal narratives conversational to sustain the task. Fictional stories presented within a stimulative context would elicit context-sensitive narratives from them. Schneider and Dubé (2005) insist that there may be variability in the narrative performance of the child according to the presentation of the stimuli.

SR is one method to elicit a narrative that involves telling a story and having him/her retell the same story in his/her own words. It comprises recollection of a story where the topic, matter, and discourse length vary across different individuals as they must extract from their lexical and linguistic skills for SR. It is seen as the best predictor of language development delays in young children as it reflects their capability to deduce and reconstruct a sequential narrative (Gazella and Stockman, 2003).

SG requires the narrator to develop a story schema in his/her own words. For a child to generate a story, he/she must produce the story sequence from scratch, with a baseline story schema, from their experiences upon seeing pictures or hearing auditory stems. The narrator must be unique in developing his/her narrative, as SG for the first-time requires the interplay of both cognitive and linguistic skills.

SR and SG require linguistic (syntactical and semantical) and pragmatic (contextual usage) skills that are fluently interwoven together (Mäkinen et al., 2014; Wood et al., 2018; Wofford and Wood, 2019). The oral narrative quality and length depend on the elicitation procedure (Schneider and Watkins, 1996). Hesketh (2004) also found evidence supporting the assertion that retelling a previously heard story is easier than creating an original, novel one. This could be because retelling a story is a comprehension-based task whereas SG is a creative task (Hansen, 1978); however, SG reflects better reflect narrative organization skills.

The quality and quantity of narratives are often organized and analyzed as two major components: (a) macrostructure components (qualitative) that describe the overall structure and content of the narrative and (b) microstructure components (quantitative) that focus on language productivity and internal linguistic elements such as clauses, conjunctions, verb forms, and nouns. The microstructure of utterances reveals syntactic and semantic productivity, complexity, and exactness of the words required to maintain cohesion. The microstructures of narratives are often calculated using the total number of words (TNW), mean length of utterances (MLU), number of different words (NDW), number of utterances, number of communication units or T-units, and type-token ratios (Reese et al., 2012). These microstructure measures are often used in language sample analysis and in other indices developed for assessing narratives. TNW in a narrative signifies the length of the story, use of vocabulary and also reflects the overall verbal fluency of the children (Leadholm and Miller, 1994). Within a narrative, the TNW and NDW distinguish children with high and low language abilities (Muñoz et al., 2003). MLU reveals the syntactic organization, with the typical number of words used to make an utterance. MLU is indeed a good indicator of a child's language development (Ranalli, 2012). MLU calculated in words/morphemes indicates a children's linguistic growth and helps us monitor the grammatical complexity in their narrative performance (Gillam and Gillam, 2016; Muñoz et al., 2003). The number of utterances is a parameter used for narrative analysis, and it increases with age (Crookes, 1990; Hoffman, 2009). NDW and TNW are measures of lexical diversity in children's narrative production, whereas the number of utterances and MLU measure their syntactic complexity (Justice et al., 2006). A composite evaluation of these microstructure measures often considered being the best predictor of age-appropriate language development in children. These narrative productivity measures are used to distinguish children with language deficiency from neurotypical children. Even though microstructure measures tend to increase with age, a quantifiable increase across age should be profiled in order to evaluate the children's narratives quality (Hoffman, 2009). Various narrative microstructure indices have been developed and standardized in English, as shown in Table 1.

**Table 1:** Various microstructure narrative indices.

	Narrative Assessment Protocol (NAP) Justice et al. (2010)	Edmonton Narrative Norms Instrument (ENNI) Schneider and Dubé (2005)	Systematic Analysis of Language Transcripts (SALT) Miller (1980)	INMIS Justice et al. (2006)	Sampling Utterances and Grammatical Analysis Revised (SUGAR) Pavelko and Owens (2017)
Age range	3 years to 6 years	4 years to 9 years	4 years to 12 years 8 months	5 years to 12 years	3 years to 7 years 11 months
Elicitation tasks	SG from a wordless picture book	SG and story comprehension	SR and SG	SG with wordless picture book	SR and conversation
Narrative elements assessed	Sentence structure, phrase structure and advanced modifiers, noun, verb, etc.	TNW, number of different words and mean length of communication units in words	TNW, MLU and type-token ratio (TTR), number of different words	TNW, mean length of T-units in words and morphemes, number of different words	TNW, MLU, words per sentence, clause per minute

Based on the narrative elicitation procedure used children's narrative performance varies, the differences and similarities are discussed below.

Mäkinen et al. (2018) followed up on the relationship of narrative and reading skills in neurotypical Finnish children in a three-year longitudinal study. Twenty children were tested on narrative retell and SG tasks twice, at the ages of five and eight. These children formed longer stories in retelling task than in the SG task. Such differences notwithstanding, both procedures aim at accessing the highest language organization abilities.

DiSegna Merritt and Liles (1989) evaluated the narratives produced by language-impaired and non-impaired children aged nine to eleven years and four months in both SR and SG tasks. In the retelling task, both groups produced longer narratives, more story components, and complete episodes. In the SG task, clause length was shorter, and episode completion was less frequent. They also found the SR task to be more compliant for clinical use. A large amount of detailing and deviation from the context of stimuli in SG task makes scoring the sample more difficult and less reliable. Given the fact that SR is often regarded as an easier task than SG, the study suggested that both were effective gauges of narrative ability and activated a cognitive organization consistent with the storey schema.

Westerveld and Gillon (2008) studied the impact of narrative elicitation on the performance of children's oral language. A group of eleven children (aged seven years eleven months to nine years and three months) with reading disabilities and a control group (age-matched) of equal number of children with age-appropriate reading skills constructed narratives in different contexts: SR, SG, and personal narratives. Microstructure measures of semantic diversity, verbal productivity and morphosyntax were examined in the study. The findings revealed no significant interactions between both the groups implying that the children responded similarly to the elicitation contexts. They reported presentation of longer narratives in SR than in SG tasks and the results showed that SR was more reliable and yielding in eliciting narratives.

Schneider and Dubé (2005) suggest that SR contexts are more effective than SG contexts in bringing about a longer and more extensive narrative sample from young children. Tonér (2019) compared SR and SG tasks among 431 typically developing Swedish children aged between three years and six years and four months. The comparison revealed that SG had more morphosyntactic correctness and syntactic complexity than SR. It also led to longer stories compared to SR conditions. However, SR was found to have more story content than SG did.

### 1.2 Tamil and children's narratives

Tamil is an ancient Dravidian language spoken in India and Sri Lanka. There are just two works published on narratives of typically developing Tamil-speaking children: Priyadharshini (2017) and Ravichandran et al. (2020).

Priyadharshini (2017) analyzed the development of story grammar elements (macrostructure) in the narratives of Tamil-speaking children between five and eight years, with 15 children in each age group. The study was carried out using the “Frog, where are you?” story, which was normed with the English-speaking population. Ravichandran et al. (2020) analyzed the syntactic and semantic diversity in self-narratives and SR among 30 Tamil-speaking children from first and second grade. Ravichandran et al. (2020) analyzed narrative development, gender difference, and task variation in the microstructure parameters, namely TNW, MLU, NDW, and type-token ratio. Children tend to narrate as early as two years of age. The early development of narrative has a considerable influence on children's subsequent language and literacy development (Heilmann et al., 2010; Justice et al., 2006).

Studies on emerging narratives in typically developing Tamil-speaking children have not been carried out yet. The tasks and materials used in these two studies aimed at assessing different genres of narratives. The narrative parameters they evaluated varied between the studies, and the sample size of thirty in each study was inadequate to generalize the findings.

Venkatraman and Vijayarangam (2020) compared the microstructure measures, NDW and MLU, of typical developing Tamil-speaking children and verbal children with autism, in the age range of six to eight years. The SR task they employed to elicit narratives reflected a reduced NDW and MLU in children with autism than those with typical development. Although the parameters reflect an inadequacy in narratives, no standard protocol and norms have been established for Tamil in order to quantify the inadequacy in narratives. Narrative measures that are time efficient, simple, and easier to calculate, score, and interpret must be established for regular clinical evaluation (Hoffman, 2009). MLU, TNW, and utterances are often used in regular language assessment too, so it would be easy to carry out the narrative assessment time-efficiently. Since these measures are common to narrative and language assessment, they would be easy for clinicians to use.

The procedural influence in elicitation of narratives has had mixed results. There is no consensus regarding appropriate procedures for eliciting children's narratives because each study reported different outcomes measures of the narratives used (Adani and Cepanec, 2019). The literature reports mixed findings on gender differences in narrative productivity, studies are reporting no significant differences between males and females (Muñoz et al., 2003; Ravichandran et al., 2020; Safwat et al., 2013), while few report females outperform males (Kaderavek et al., 2004). An insight into developmental changes in narrative elicitation procedures would streamline the evaluation and intervention protocols for children with language disability. As the inadequacy of established normative data for microstructures of narratives in Tamil-speaking preschool children is lacking as mentioned above, this study takes into account three empirical microstructure parameters which are regularly used in clinical language evaluation.  

***Objectives***

1. To record the developmental trends in SR and SG tasks across four groups of Tamil-speaking children aged three years to six years and eleven months (Table 2).
2. To compare the effect of elicitation context on the microstructure elements of narratives across these age groups.
3. To find whether there are any gender differences in microstructure parameters in the SR and SG contexts.

**Table 2:** Demographic data of participants.

Age (Years; Months)	Total Number of Participants (n = 200)	Male/Female	Mean Age (Years; Months)
3–3; 11	50	15/15	3; 7
4–4; 11	50	15/15	4; 6
5–5; 11	50	15/15	5; 5
6–6; 11	50	15/15	6; 6

## 2 Methodology

### 2.1 Participants

The sample consisted of 200 typically developing three years to six years and eleven months old preschool as well as primary school children from eight schools in Chennai who used Tamil as the primary language. Participants were assessed for speech, language skills, and hearing ability. Children with speech delays, sensory difficulties, or late language development were screened out. The participants were recruited based on convenient sampling and demographic/personal data were collected for every participant. The children were classified into four age groups, with an identical number of boys and girls in each group, as shown in Table 2. The study was conducted according to the established ethical guidelines of the Annamalai University, Chidambaram. Prior to participation, each participant's parents were explained about the study thoroughly and informed consent from them is secured.

### 2.2 Material

The stimulus used for the SR and SG tasks was evaluated for its content which was checked by two linguists, five preschool teachers, and a counsellor. A pilot study on the familiarity of stimulus for both the task was conducted with ten children from the first two groups. The stimulus used for SR was selected from storyweaver.org,^1^ a website where stories are categorized age-wise. “My fish, no fish” was the most familiar story to the children and was used as the stimulus. The story had colourful pictures and Tamil text.

SG was conducted using “What is next – level 1” from creative educational aids (Appendix A). The original material had eight sets of picture-sequencing cards, with four cards in each set. Out of the eight sets, five were used as test stimuli, two were used to demonstrate the task, and one was removed due to unfamiliarity. The lower age group cannot generate stories if there is too much structural complexity in the study; therefore, the task was simplified based on the pilot study. The time allotted to narrate the stimuli selected for both SR and SG tasks was equal. The content of both stimuli could not be equated as there are no standard scripts for SG.

### 2.3 Story elicitation

The study participants were instructed to look at the colourful pictures, pay close attention to the researcher's narration of the story, and repeat it when asked after a two-minute break while viewing the storybook. A sample story sequence was demonstrated to elicit SG. They were encouraged to generate stories after seeing the prearranged sequence of four cards. Speech samples for both tasks were audio-and video recorded and transcribed verbatim. The recording duration for both the tasks was approximately three minutes. Before beginning the tasks, a rapport was created with each participant. If the child had difficulty narrating during either of the tasks, a maximum of five neutral prompts were provided such as afterwards, anything more, like, that is … After completing the task, participants were given a toffee as a reward.

### 2.4 Analysis and transcription

For the analysis of microstructural elements, all utterances were included, except the researcher's neutral prompts, mazes, false starts, and repeated utterances of the children. The transcriptions were first marked for utterances and then analysed for the constituent microstructural elements for both SR and SG tasks, namely MLU, TNW, and the number of utterances.

1. TNW was computed by counting the number of words in each sample after removing mazes, false starts, and repeated utterances (Justice et al., 2010).
2. The number of utterances (see: Crookes, 1990).was calculated by demarcating the utterance and counting them.
3. MLU was computed by dividing the number of utterances by TNW (Baixauli et al., 2016).

A quarter of the sample was randomly selected to measure the inter-rater reliability of the data's parameters. The reliability measured using Cohen's Kappa reflected 92% inter-rater reliability between the two raters. The results were tabularized in MS-Excel and grouped age-wise. The tabulated data were then analysed using SPSS (version 21) to examine the effect of the narrative context, developmental trends, and gender differences across the groups.

## 3 Results

### 3.1 Developmental trends in the narrative context

The developmental trends in SR and SG of narratives in Tamil-speaking children between three years and six years and eleven months were analysed in terms of the microstructural components of narratives. Three parameters were assessed to measure language productivity, namely, TNU, MLU, and number of utterances. The age-wise descriptive data of the three parameters were calculated across four age groups. The mean and standard deviation of the parameters are described in Table 3. The results revealed a significant increase in all three parameters of microstructure elements in both the SR and SG contexts.

**Table 3:** Mean and standard deviation (SD) of the TNW, MLU and the number of utterances obtained from SR and SG tasks.

Narrative Context/Age	Story Retelling						Story Generation					
	TNW		MLU		Number of Utterances		TNW		MLU		Number of Utterances	
	Mean	SD	Mean	SD	Mean	SD	Mean	SD	Mean	SD	Mean	SD
3 years	53.38	4.40	3.48	.504	16.08	1.65	41.88	1.42	3.30	.46	13.20	.70
4 years	69.24	3.19	4.44	.501	14.58	1.69	61.88	12.09	4.23	.42	14.90	1.34
5 years	86.88	3.42	5.40	.484	16.16	1.07	81.50	3.02	4.78	.42	17.08	.75
6 years	113.14	11.80	6.40	.571	18.28	1.76	94.98	2.53	6.00	.40	16.44	1.07

ANOVA was used to analyse the development of narratives across the four age groups for the three parameters (Table 4). There was a significant difference in TNW, MLU and number of utterances, as F value (3, 196) = 726.49, p-value <0.001 for TNW, F value (3, 196) = 295.09, p-value <0.001 for MLU, and F value (3,116) = 46.99 with p-value <0.001 for the number of utterances was obtained in SR. SG also revealed a significant difference across the four age groups, as F value (3, 196) = 656.47, p-value <0.001 for TNW, F value (3, 196) = 349.44, p-value <0.001 for MLU, and F value (3, 196) = 149.52, p-value <0.001 for the number of utterances was obtained.

**Table 4:** Comparison of microstructure elements of narratives in the SR and SG.

Group	Story Retelling		Story Generation	
	F	*Sig*	F	*Sig*
TNW	726.49	<0.001∗	656.47	<0.001∗
MLU	295.09	<0.001∗	349.44	<0.001∗
Number of utterances	46.99	<0.001∗	149.52	<0.001∗

∗p-value.

Owing to significant differences in ANOVA, a post-hoc test for multiple comparisons was used to assess the difference betweenthe groups on parameters TNW, MLU, and number of utterances in SR and SG (Table 5). The Benforni post-hoc pair-wise comparison indicated a significant difference between the groups in TNW with the p-value <0.001 in SR and SG. The results revealed an increasing trend in this parameter as the age increased in both the SR and SG contexts. MLU also reflected a significant difference across the four age groups, with the p-value < 0.001 in SR the two contexts. MLU showed a steady increase across the groups in both SR and SG. There is a significant difference in the number of utterances in both the elicitation contexts across the groups, with the p-value < 0.001. However, no significant differences were noticed among three- and five-year-old children in SR, as the p-value (1.00) is greater than 0.05.

**Table 5:** Post-hoc comparison of microstructure elements of narratives across the groups in SR and SG.

Post-Hoc Comparisons						
Group	Story Retelling			Story Generation		
	Mean Standard Error	Sig	Results	Mean Standard Error	Sig	Results
TNW	1.34	<0.001∗	3 years < 4 years <5 years < 6 years	1.28	<0.001∗	3 years < 4 years <5 years <6 years
MLU	.1033	<0.001∗	3 years < 4 years < 5 years < 6 years	.0853	<0.001∗	3 years < 4 years <5 years <6 years
Number of utterances	.314	<0.001∗	3 years < 4 years<6 years	.200	<0.001∗	3 years < 4 years<5 years < 6 years

∗p-value.

### 3.2 Story retelling versus story generation

The variability in narrative performance in SR and SG was assessed across the four groups. An independent sample t-test was conducted to contrast the parameters elicited in both contexts. TNW of children in SR (M = 80.66, SD = 23.217) was more significant than that in SG (M = 70.06, SD = 21.109, t (398) = 4.777, p-value <.001). The MLU of children in the SR (M = 4.930, SD = 1.2041, p-value = 0.001) was more significant than SG (M = 4.578, SD = 1.067, t (398) = 3.09, the p-value = .002). The number of utterances of children in SR (M = 16.28, SD = 2.042, p-value = 0.001) was more significant than SG (M = 15.41, SD = 1.802, t (398) = 4.51, p-value <.001). Therefore, the independent-sample t-test (Table 6) revealed more productivity in the SR context in all three parameters of the microstructural elements than in the SG context.

**Table 6:** Comparison of SR versus SG using Independent sample t-test.

Parameters	T	df	Sig. (2-tailed)	Mean Difference	Std. Error Difference
TNW	4.77	398	<.001∗	10.60	2.22
MLU	3.09	398	.002∗	.352	.11
Number of utterances	4.51	398	.001∗	.870	.19

∗p-value.

### 3.3 Gender differences

Gender differences in the narrative performance for both elicitation tasks and microstructural parameters were analysed for each age group. Mann-Whitney U-test was employed to compare ‘girls’ and boys' performances in each group (Table 7).

**Table 7:** Gender difference in SR and SG across the groups using Mann-Whitney U-Test.

Age Groups		Story Retelling			Story Generation		
		TNW	MLU	Number of Utterances	TNW	MLU	Number of Utterances
Three years	Mann-Whitney U	259	312.5	269.5	301.50	225	196.5
	Wilcoxon W	584	637.5	594.5	626.5	550	521.5
	Z	-1.058	.00	-.85	-.22	-2.14	-2.45
	Asymp. Sig. (2-tailed)	.290	1.0	.392	.82	.032	.014
Four years	Mann-Whitney U	210.5	287.5	306.5	276	309.5	302.5
	Wilcoxon W	535.5	612.5	631.5	601	634.5	627.5
	Z	-1.99	-.564	-.118	-.722	-.078	-.208
	Asymp. Sig. (2-tailed)	.046	.573	.91	.47	.94	.84
Five years	Mann-Whitney U	293.5	206	195	266.5	300	308
	Wilcoxon W	618.5	531	520	591.5	625	633
	Z	-.37	-2.38	-2.39	-.906	-.338	-.095
	Asymp. Sig. (2-tailed)	.71	.017	.017	.365	.735	.924
Six years	Mann-Whitney U	308.5	183.5	235	292	289.5	286.5
	Wilcoxon W	633.50	508.5	560	617	614.5	611.5
	Z	-.078	-2.980	-1.533	-.403	-.700	-.529
	Asymp. Sig. (2-tailed)	.938	.003	.125	.687	.484	.597

The Mann-Whitney U-test indicated that three-year-old boys and girls showed no difference in story length, as TNW was not significantly different in SR (U = 259, p-value = .290) and in SG (U = 301.5, p-value = .826). The U score and the level of significance obtained for MLU in SR was U = 312.5, with the p-value of 1.000, while in SG, it was U = 225, with the p-value of .032. The number of utterances revealed the U-score of 269.5 with a p-value of .392 in SR and 196.500 with the p-value of .014 in SG. SR did not reveal a significant gender difference for all three parameters. MLU and the number of utterances in SG reflected that girls performed better than boys.

TNW of the four-year-old girl children reflected a better narrative performance, with the U score of 210.5 and p-value of 0.046 in SR. Four-year-old girls produced more words in than four-year-old boys in SR.

Compared to five-year-old boys, five-year-old girls showed a higher MLU, with a score of U = 531 and a p-value of 0.017, and number of utterances with a score of U = 195 and a p-value of 0.017 in the SR.

Six-year-old girls display increased scores on MLU (U = 183.5, p-value = .003) and number of utterances (U = 61.000, p-value = 0.029) in the SR context compared to six-year-old boys.

## 4 Discussion

### 4.1 Developmental trends

The increase in TNW with an increase in age was similar to that obtained by Khan et al. (2016), who examined the narratives of 386 English-speaking children between three and six years. Tilstra and McMaster (2007) measured the TNW produced by 45 kindergarteners, first-graders, and third-graders, and found that TNW increased significantly with age.

The increase in TNW could be attributed to the children's ability to make hierarchical relationships between events in a complex narrative production as age increases (Heilmann et al., 2010). TNW signifies the story length, which becomes longer and richer as children can evaluate their own stories into their verbal performance (Muñoz et al., 2003). The richness in the TNW is related to the acquisition of new vocabulary through repeated exposure to narrative forms through the preschool and young school-age (Heilmann et al., 2010). The gradual increase in TNW is coherent with the typical language development. The rate of acquisition of vocabulary is higher in younger ages, peaking at school-going ages (Nóro and Mota, 2019). The rapid increase in the gaining of new vocabulary is reflected in the narrative ability of children across both the elicitation tasks.

The ability to create a longer chain of events and recall the events coherently has implications on story length. As a result, this microstructure metric could reveal information about a child's overall narrative productivity. As a measure of story length, TNW appears to reflect language output and improves with both language skill and chronological age (Justice et al., 2006).

MLU has been a valuable measure in assessing language development (Rice et al., 2010). It is an indicator of the syntactic complexity involved in children's utterances as it shows a steady increase with age and reflects age-related syntactic complexity. The mean length of utterance showed a steady increase with an increase in age and thereby reflected age-related syntactic complexity. The current study reiterates the existing findings that MLU is an index of linguistic maturity and grammatical development (Ranalli, 2012). The semantic and syntactic role of MLU, as evaluated by Brown (1973), clearly denotes that when children reach an MLU of more than three morphemes, they tend to have coordinated sentences. Children predominantly use content words at two years of age, and from three to five years old, they gradually add functional and grammatically complex words to form longer sentences (Nóro and Mota, 2019). Almost every specific component of linguistic knowledge that children acquire lengthens their uttered sentences. Therefore, the acquisition of words or vocabulary is a requisite and a critical aspect of syntactic development.

The current study substantiates this assertion as TNW and MLU both show concomitant increases across the four age groups in both narrative elicitation tasks (Nóro and Mota, 2019). The number of words used in a sentence also signifies the children's vocabulary and its access from the semantic memory. Ravichandran et al. (2020) reported similar trends in six to eight-year-old, neuro-typical urban Tamil speaking children in self-narration and SR context.

The number of utterances as an index of language productivity showed a gradual increase across the four age groups in this study. The outcomes of this work reinforce the observations of Muoz et al. (2003), which reveal that three-year-old can create two or three events in a narrative, four-year-old can create distinct sentences connected to the story, and five-year-old can create interrelated sentences. Children's ability to use subordinate clauses develops from their preschool years and continues throughout their school years (Heilmann et al., 2010).

However, there was no significant difference in the number of utterances between the age groups three years and five years in the SR context. Children depend on their vocabulary skills before they acquire complex syntax to organize narrative production. The acquisition of vocabulary and its use in narrative production is evident from the steady increase in the parameters of TNW and MLU with age. However, the number of utterances parameter which is related to the syntactic development could reflect an overlap in developing narratives of children (Heilmann et al., 2010). In terms of semantic and syntactic patterns of narratives, there is a range of overlap between grades, and a similar pattern of narration may be noticed between children of different grades (Johnson, 1995). The narrative turns sophisticated and gains complexity from five years of age. The children at this age describe and express almost 73 % of story structures in their narratives (Hedberg and Stoel-Gammon, 1986). The cognitive load to match story structures of the adult in a retell might restrict the utterances number. This variation was not noted in the SG task as it reflects the genuine narrative skills of the children. As the complexity of the macrostructures increases, there is a tendency for the microstructure elements to decline in quantity, the trade-off mentioned in the literature was observed in the present study (Justice et al., 2006). However, the six-year-olds have more utterances; than five-year-olds, this reflects the sophistication in narrative skills acquired by this age.

The developmental trends tend to be evident in both tasks, despite that it taps two different aspects of narratives, that is SR is more of a comprehension process while SG is an expressive process. The developmental change could be due to factors such as familiarity with the content, experiential knowledge of elements in the story, story complexity, and interpretative skills of children. Familiarity of the content is better in the older age group than the lower age one, which is consistent with the developmental changes observed in this study. The complexity of both the generated as well as retold story could also be attributed to these changes. The length and number of episodes added to a story impacts retelling and generation of the narrative. The lower age group show a limited capacity to handle a chain of events in a story. The comprehension skills of a child are important in retelling the story. Preschoolers are not mature enough to interpret and name all the elements of the story, while the older age group is sensitive to story settings and personal motivation of the characters in the story (Berman, 1995).

### 4.2 Story retelling versus story generation

Language productivity is observed to be more in SR than in SG. This finding is coherent with the studies quoted in the literature (Mäkinen et al., 2014; Westerveld and Gillon, 2010a).

All three parameters of narratives used in the study reflected more productivity in SR than in SG. This difference in performance between the two tasks could be attributed to the conceptual development that occurs due to socialization, which in turn accelerates the child's internalization from a Vygotskian view (Schneider and Watkins, 1996). The narrator's pre-modelled narration creates an internalization of the story schema, thereby increasing the productivity in the SR context. SG from scratch from a picture or from an auditory stimulus is a difficult and demanding task (Westerveld et al., 2004). Peterson and McCabe (1994) argue that children tend to be confused and have difficulty finding words as they create stories based on picture stimuli.

The narrative task seems to employ integration of cognition and memory in the most logical order. Thus, the performance difference in SR and SG has to be explained from cognitive and memory correlates of language alongside the behavioural understanding. The SR task is often thought to be a comprehension process, as a similar model of the story is given to the children. Retelling is a top-down process that occurs in recognizing matching narrative patterns. In contrast, generations happen by a bottom-up process of evaluating the received sensory input from the stimuli and framing that in a story schema (Anderson, 2015).

The bucket theory argues about performance trade-offs across distinct language tasks and explains the variance in performance across two narrative elicitation tasks based on cognitive loads (Crystal, 1987). When retelling a story, children find it easier to use structural support provided by the narrator's model, which is evident from the improved performance in all three microstructure parameters when compared to the generation task. The need for creation and planning of a fictional story in SG tasks may require a greater cognitive strain on children than SR task. As the complexity of the language task increases, there is a reduction in the microstructural parameters of narratives in children.

León (2016) also explained a cognitive architecture for narratives and considered the term *narrative memory* to be a subset of episodic memory and semantic memory. Narrative units are stored as chunks and retrieved from the narrative memory, which reflects the integration of episodic and semantic memory. The ability to chunk the information could be the reason for the developmental increase in narrative parameters and which increases with age, reflecting a gradual change in narrative tasks’ observed performance.

**Figure 1:**
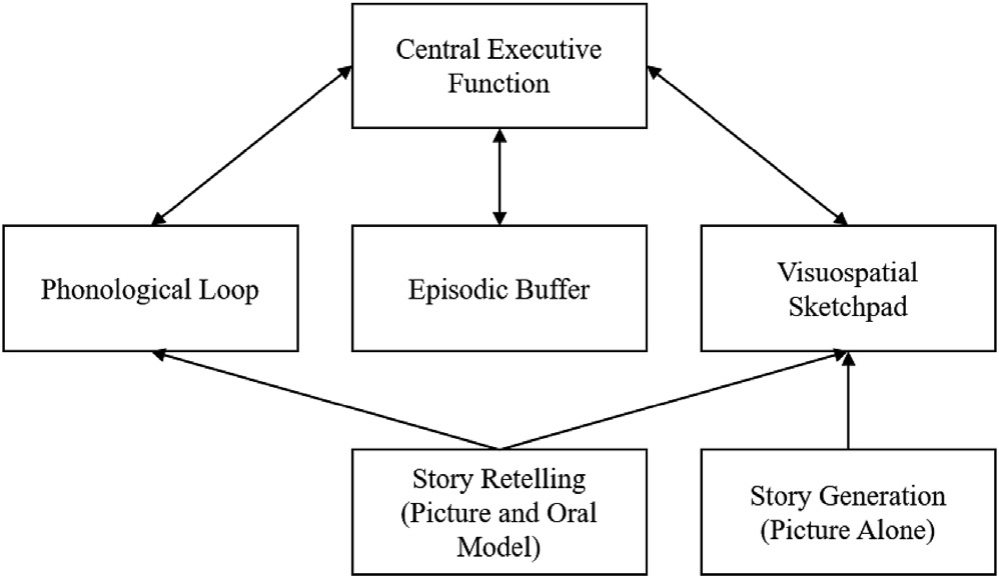
Baddeley (2000) model of working memory and its components adapted to explain narrative elicitation tasks. *Note*. From “The episodic buffer: A new component of working memory?” by Baddeley (2000), Trends in Cognitive Sciences, 4/11, p. 417–423. Copyright 2000 by Copyright Holder. Reprinted with permission.

Baddeley's model of working memory could be applied to explain the performance difference in narrative tasks. The primary components of the model are a phonological loop that stores verbal information from the ongoing speech, a visuospatial sketch pad that processes visual and spatial information, an episodic buffer that corresponds to the sequential organization of events, and a central executive structure that collates the information between these components and long-term memory (Figure 1). SR involves the narrator to verbalize the storyline, describe the picture sequence to the children, and later ask them to describe the picture sequence. In contrast, SG task is elicited by the mere presentation of visual stimuli like the picture sequence used in the present study. Therefore, it could be attributed that the retelling task activates visuospatial sketch pad and phonological loop, making it an auditory-verbal input. However, SG that exclusively involves visual stimuli presentation cannot tap into the phonological loop directly. Hence, the SG task could activate only the visuospatial sketch pad component to process the visual stimuli. The activation of these two components simultaneously could be the reason for improved performance in a SR than in a SG. The Baddeley (2000) model of working memory also suggests that a task with dual-modality stimulation tends to be more efficient than a single modality. When a narrative elicitation task has a dual-modality of stimulus presentation, the narratives tend to be dense, as seen in the SR task.

The use of the auditory-verbal mode of stimuli would be crucial while assessing young children, as they might apply the cues of one modality to other modalities to complete the task. SG seems to reflect the complexity involved in processing unisensory stimuli, reflecting the children's genuine ability to produce a self-made narrative.

SG task gets more sophisticated from the age of six years onwards. Children by this age can retell and comprehend canonical stories well. Childrens’ increased complex cognitive capability enables them to express and comprehend more complex story material (Miles and Chapman, 2002). The story schema to construct narratives seems to get more comprehensive from six years of age.

The present study tries to understand the difference in performance between narrative elicitation tasks based on the intrinsic cognitive and memory processing that happens on exposure to narrative stimuli. The results highlight the expected performance variation in the elicitation procedure used during the narrative assessment of children with language deficiency and the intervention process for language therapy. The results suggest that while assessing pre-schoolers, the choice of eliciting narratives should be a SR condition. However, the complexity of assessing a narrative could be augmented by a SG task for children older than five years to find their ability to construct a narrative (Hoffman, 2009; (Pavelko and Owens, 2017); Shiro, 2003). This suggestion is made with the notion that comprehension precedes expression since retelling is a comprehension-based task younger children would be able to perform narratives, therefore avoiding underestimation of their narrative skill.

### 4.3 Gender difference in narratives

Gender differences were not uniformly observed across the parameters in the elicitation task in this study. Three-year-olds exhibited differences in MLU and the number of utterances in the SG task. Four-year-olds showed differences in TNW in a SR task, while five and six-year-olds showed differences in MLU and the number of utterances in the SR task. This difference from the Mann–Whitney U-test shows better performance by girls in certain parameters and contexts as compared to the boys.

The early onset of clauses in the spontaneous speech of girl children could explain the differences in MLU and the number of utterances in three-year-olds on SG and SR (Adani and Cepanec, 2019). In the initial few years of their life, girls’ lexical and grammatical development tend to be more rapid. Boys produce word combinations three months later than girls, according to Adani and Cepanec (2019), which can also explain the difference in MLU and the number of utterances between boys and girls. The gender differences in certain parameters could be due to the early acquisition of language, innate rapid vocabulary acquisition, and its presentation in social communication context possessed by girl children Adani and Cepanec, 2019.

The literature suggests that not only gender differences but also variables such as age and nature of task influence linguistic performance (Justice et al., 2006). Ravichandran et al. (2020) observed no significant gender difference in the narrative performance of Tamil speaking children in the age range six years to eight years in the microstructure parameters evaluated. Also, the typical development of narratives in Arabic-speaking European children between two and six years revealed no significant gender differences (Safwat et al., 2013). However, the outcomes of the study cannot be generalized as a gender difference due to the inconsistency in its presentation across age groups and elicitation tasks.

## 5 Conclusion

The present study aimed at identifying the developmental trends, effect of narrative elicitation context, and effect of gender difference on the narratives by Tamil-speaking children. Although there are many microstructure parameters, this study focused on TNW, MLU, and the number of utterances because these conventional measures can be calculated by hand. These measures make narrative assessment convenient as the parameters are regularly used in clinical language assessment. These parameters measure the semantic and syntactic complexity of the narrative (Justice et al., 2006).

Although there are several language sample analysis methods, the practical problem with its clinical usage is time constraints and multiple parameters for the calculation. An age-wise criterion-referenced measure developed with conventional language assessment measure would solve the time constraint issue faced during a narrative assessment (Pavelko and Owens, 2017). These metrics could help us understand the baseline narrative skill of a child with language disorder and set goals during language therapy for children with a language disorder. As the narrative skill requires cognition, memory and language, even after a continuous narrative intervention, if the child does not show any progress in these metrics, it would direct to evaluate detailed cognitive and memory skills to address the language inadequacy.

Several studies show a similarity in the acquisition of vocabulary in children across different language groups. Although there are similarities in the acquisition pattern in these narrative micro measures, the quantitative measure varies across languages and tasks (Shiro, 2003). Therefore, it is important to develop normative data for every language. The literature comparing narratives of neurotypical children, children with autism, ADHD, specific language impairment, Down's syndrome, and reading disability consistently report a quantitative decrease in the microstructural measures like TNW, MLU and number of utterances (Baixauli et al., 2016; Feagans and Appelbaum, 1986). These studies also emphasise performance variation in SR and SG tasks. Results from this study also support the finding that SR is more productive than SG. The interplay of working memory and cognitive abilities alongside linguistic abilities has been noted and explained as the reason for differences in narrative performance between typically developing children and those with language disabilities.

This study also has implications in language therapies for children with language disorders. Even after they start speaking, children with language impairment tend to exhibit inadequate narrative skills. The quality of the narratives they produce can be evaluated and goals could be framed to improve the inadequacy in their language. Gender differences could not be generalized as there are inconsistencies in its presentation across the age groups, parameters, and contexts. Narrative analysis in the literature shows that language sample analysis is more time consuming and often not practised in a clinical scenario (Pavelko and Owens, 2017). Although several indices are used to measure narrative productivity in typically developing children in various languages, there are no such data for Tamil.

This study addresses the prime need to establish normative data in typically developing Tamil-speaking children. It considered convention measures common to language evaluation and microstructures of narratives to make the data clinically useful. These measures reflect a quantitative difference in typical narrative development with age and progression in language skills. The current study helps to identify age-appropriate narrative behaviors in Tamil speaking children and provide directives to avoid incorrect attributions of the developmental process. They would help in setting various criteria during language therapy and also in monitoring the progress. Further studies on other semantic and syntactic categories like nouns, pronouns, tenses, and adjectives and their distributions would help us relate the development of the internal construct of narratives in children.

## Declarations

### Author contribution statement

Krupa Venkatraman: Conceived and designed the experiments; Performed the experiments; Analyzed and interpreted the data; Contributed reagents, materials, analysis tools or data; Wrote the paper.

V. Thiruvalluvan: Contributed reagents, materials, analysis tools or data.

### Funding statement

This research did not receive any specific grant from funding agencies in the public, commercial, or not-for-profit sectors.

### Data availability statement

Data will be made available on request.

### Declaration of interests statement

The authors declare no conflict of interest.

### Additional information

No additional information is available for this paper.

## Footnotes

1 https://storyweaver.org.in/stories/49-my-fish-no-my-fish.
