# Supplementary material for: Development of narratives in Tamil-speaking preschool children: A task comparison study
Source: Heliyon. 2021 Jul 21;7(7):e07641. doi: 10.1016/j.heliyon.2021.e07641 (PMC8326734; doi:10.1016/j.heliyon.2021.e07641)
Supplement: Supplementary file 1 — Appendix [file mmc1.docx]

**Appendix A**


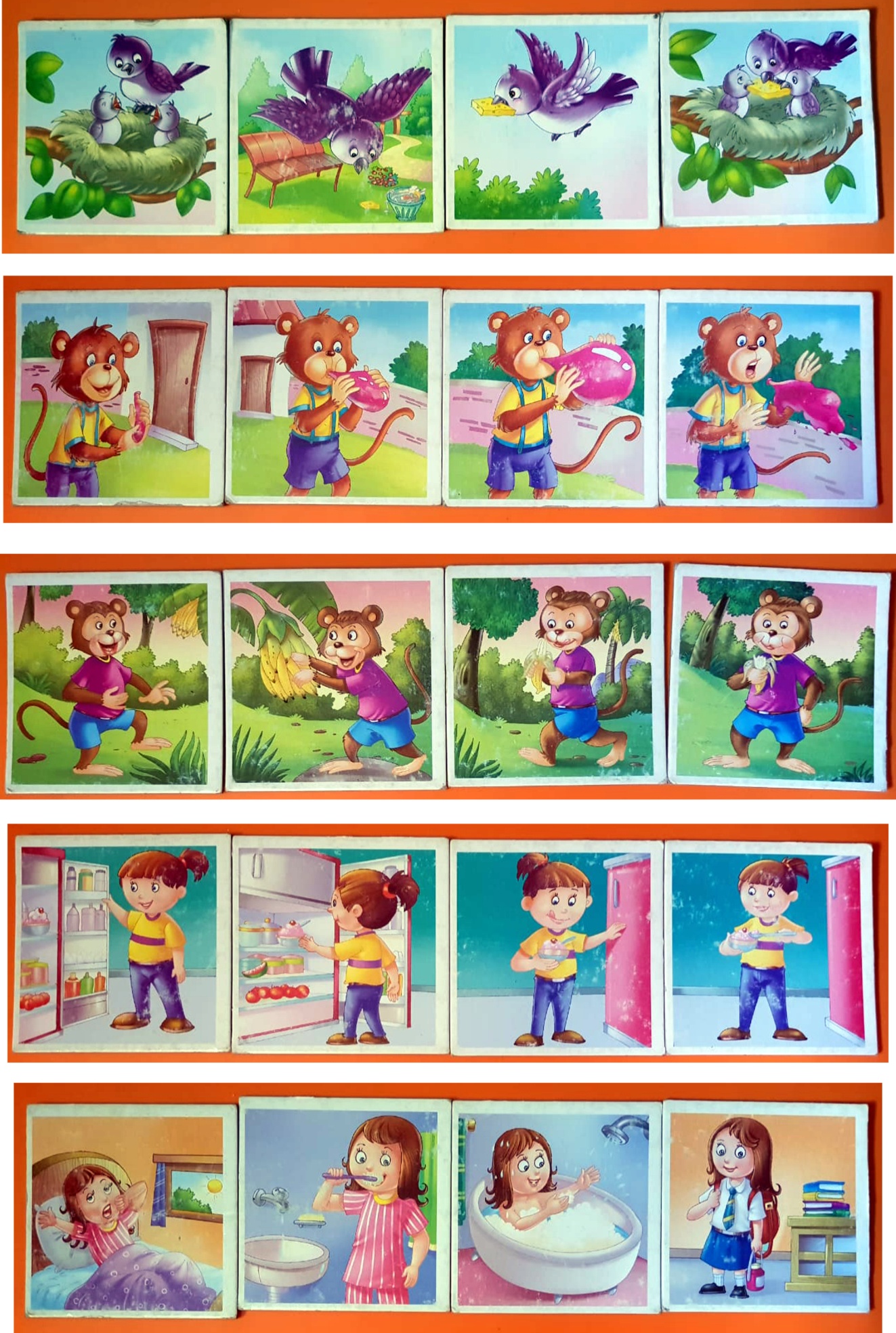
**Stimuli for Story Generation Task**

**Source:** Creative Educational Aids P. Ltd. What’s Next level - 1 Card Game

*Note.* Reprinted with permission from Creative Educational aids Pvt ltd.
